# Cartilage Oligomeric Matrix Protein Induced Arthritis—A New Model for Rheumatoid Arthritis in the C57BL/6 Mouse

**DOI:** 10.3389/fimmu.2021.631249

**Published:** 2021-02-23

**Authors:** Yunjuan Zhao, Vilma Urbonaviciute, Bingze Xu, Weiwei Cai, Zeynep Sener, Changrong Ge, Rikard Holmdahl

**Affiliations:** ^1^ Department of Endocrinology, The Second Affiliated Hospital of Guangzhou Medical University, Guangzhou, China; ^2^ Medical Inflammation Research, Department of Medical Biochemistry and Biophysics, Karolinska Institute, Stockholm, Sweden

**Keywords:** rheumatoid arthritis, cartilage oligomeric matrix protein, C57BL/6 mice, T cell epitope, B cell epitope

## Abstract

The most commonly used strains in experimental research, including genetically modified strains, are C57BL/6 mice. However, so far, no reliable model for rheumatoid arthritis is available, mainly due to the restriction by the MHC class II haplotype H-2^b^. Collagen-induced arthritis (CIA) is the most widely used animal model of rheumatoid arthritis, but C57BL/6 strain is resistant to CIA because there is no collagen II peptide associated with H-2^b^. To establish a rheumatoid arthritis model in C57BL/6 mice, we immunized C57BL/6NJ (B6N) mice with human cartilage oligomeric matrix protein (COMP), which induced severe arthritis with high incidence, accompanied by a strong auto-antibody response. Native COMP was required, as denatured COMP lost its ability to induce arthritis in B6N mice. An immunodominant COMP peptide was identified as the key T cell epitope, with a perfect fit into the A^b^ class II peptide binding pocket. A critical amino acid in this peptide was found to be phenylalanine at position 95. Recombinant COMP mutated at position 95 (COMP_F95S) lost its ability to induce arthritis or a strong immune response in the B6N mice. In conclusion, A new model for RA has been established using C57BL/6 mice through immunization with COMP, which is dependent on a COMP specific peptide binding A^b^, thus in similarity with CIA in A^q^ expressing strains.

**Graphical Abstract:**
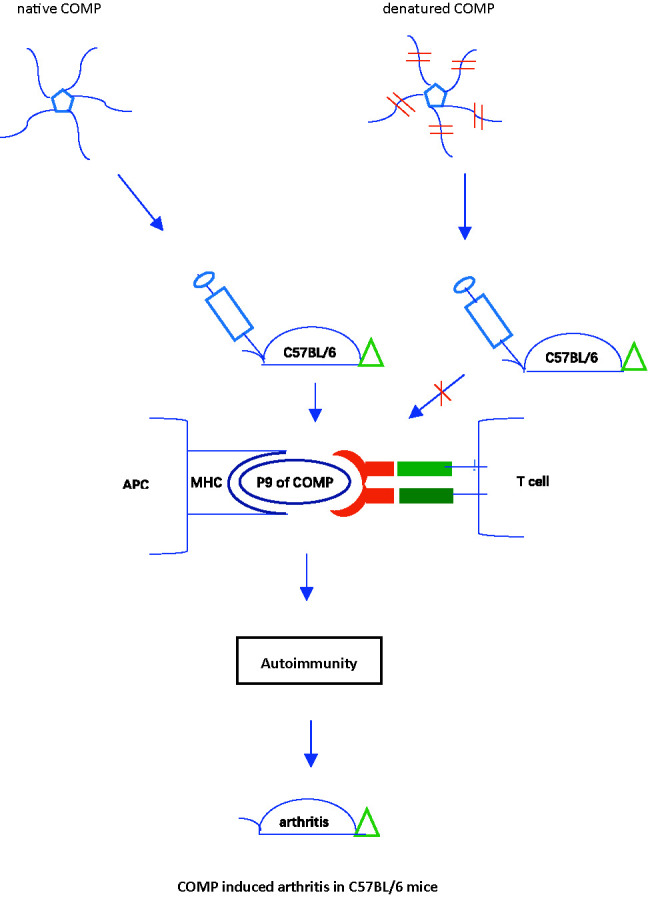


## Highlights

1. A new model of rheumatoid arthritis in C57BL/6 mice, the COMP induced arthritis (COMPIA), was established.
2. The major T and B cell epitopes on COMP critically involved in the arthritis development were defined.

## Introduction

Rheumatoid arthritis (RA) is a chronic inflammatory disease, which is characterized by leucocyte infiltration into synovial tissue and leads to cartilage and bone damage (1). It is a disease induced by not yet defined environmental factors but it also has a genetic component. The major histocompatibility complex (MHC) region class II shows the by far strongest association, strongly implicating peptide binding to an MHC class II molecule to be a critical bottleneck for the development of RA (2–5). Disease-associated risk alleles in DRB1 may share common amino acid sequences in the peptide-binding groove, suggesting peptide presentation to T cells in disease susceptibility (6, 7). To study the pathogenesis of RA, it is important to use animal models which mimic RA genetically, through an induced autoimmune response associated with the MHC class II locus.

Collagen-induced arthritis (CIA) is the most widely used animal model of RA. The susceptibility to arthritis after immunization with heterologous type II collagen (CII) is strongly associated with MHC class II genes, more precisely the A^q^ beta gene (8) due to binding of the immunodominant CII-derived glycosylated peptide 260-271 (9). In a screen of different MHC congenic strains only mice expressing A^q^ and A^r^ developed arthritis whereas mice with the A^b^ (H-2^b^) haplotype, as in commonly used C57BL/6 strains, were resistant to CIA (10–12). However, the most commonly used mouse strains in research are based on C57BL/6 (H-2^b^) (13–15), in particular in genetic research as it is often the reference standard. The discovery that immunization with various collagen preparations together with high content of mycobacteria adjuvant could induce arthritis also in B6 mice (13), has therefore been very popular and is today the most commonly used model for RA. However, despite its name, it does not represent collagen induced arthritis, as the disease develops in response to contaminants in the CII preparations (13). One contaminant is pepsin used for the cleavage of CII during the preparation and others are non-self-matrix proteins when derived from cows or chicken. It means that the T cell response binds to non-collagen proteins forming complexes with CII, enabling interaction with CII specific B cells, and thereby a specific pathogenic antibody response to CII could be elicited (16).

To establish a model for RA in C57BL/6 mice we turned our interest to other proteins that have earlier been shown to induce arthritis in mice, i.e. glucose-6-phosphoisomerase (GPI) (17) and cartilage oligomeric matrix protein (COMP) (18). We found that immunization with COMP led to severe arthritis in C57BL/6 mice.

COMP, also known as thrombospondin 5, is a pentameric glycoprotein of 524 kD (19) with five identical subunits (19). It is primarily found in cartilage, tendon, synovium and meniscus. COMP stabilizes the matrix and acts as a catalyst in the fibril formation of collagens I and II (20). It is also considered to be involved in pathological changes of cartilage during the pathogenesis of osteoarthritis (21). Immunization with COMP induces severe arthritis mimicking RA in both rats and mice (COMPIA) (18, 22). In similarity with CIA, the induction of COMPIA requires break of self-tolerance, and recognition of peptides on non–self-COMP by T cells, although the immunogenic epitopes have not been defined. Previously, the COMP induced arthritis was shown to be associated with MHC class II haplotypes, H-2^q^, H-2^p^, and also with transgenic human DR*0401 (18). However, in these studies the responsiveness of B6 mice with H-2^b^ was not included and there are no other studies describing COMPIA in C57BL/6 mice.

In the present study, we describe arthritis induced in C57BL/6 mice and identify the key COMP peptide recognized by T cells.

## Materials and Methods

### Mice

Male C57BL/6NJ (denoted B6N) mice, which carry the H-2^b^ haplotype, originally obtained from Jackson Laboratory, were used in the experiments. Animals were bred and maintained in the animal facility of Medical Inflammation Research, Karolinska Institute, with specific pathogen-free (SPF) conditions and individually ventilated cages contained wood shavings and folded paper strips. The cages were kept in the same room with a climate-controlled environment in a 12-h light-dark cycle. All of the experiments were carried out when the mice were 9–12 weeks old. Experimental mice were divided randomly, blinded and age-matched. Clinical evaluation of arthritis was performed blindly and double-checked for each experiment. All experiments were approved by Stockholm regional ethics committee for animal research, Sweden (N35/16).

### Antigens

#### Native Recombinant Human COMP

The COMP sequence was obtained from UniProtKB with accession number P49747. The construct contains the native leader sequence and the full-length COMP sequence and the C-terminal his-tag. The gene was synthesized at Eurofins with KpnI and XhoI restriction sites at the 5’ and 3’ ends. The synthesized gene was restriction enzyme digested using FastDigestTM enzymes (ThermoFisher Scientific). The digested DNA fragment was cloned into a mammalian expression vector pCEP4 (Life technologies) that was digested using the same restriction enzymes. After sequence verification, the recombinant plasmid was transfected into Expi393FTM cells (Life technologies) using the FectoPROTM DNA transfection reagent (Polyplus transfection). The supernatants were harvested 6 days post transfection. The recombinant protein was first captured using a 5 ml HisTrap Excel (GE Healthcare Life Sciences) affinity column followed by size exclusion chromatography on a HiLoad Superdex 200 pg column (GE Healthcare life Sciences) equilibrated in PBS. The purified recombinant protein (**Supplement Figure 1**) was obtained as a single peak and was concentrated using an Amicon centrifuge device with MWCO of 30 kD. The protein concentration was determined using absorbance at 280 nm.

#### Denatured COMP

Denatured COMP was prepared by incubating native human COMP in a high concentration of guanidinium chloride as previously described (23).

#### Synthetic Peptide Library of Human COMP

Overlapping peptide libraries consisting of 18-aa-long peptides (totally 76 peptides) were generated using the sequence of human COMP. Synthetic peptides containing interest sequences were synthesized by Biomatik Corporation (Ontario, CA).

#### Synthetic Peptides (P9, P9-1, P9-2, P9-3, P9-4, mP9)

Synthetic peptides (P9, P9-1, P9-2, P9-3, P9-4, mP9) were synthesized by Hangzhou Taijia Biotech Co., Ltd (Hangzhou, China). Further details are given in **Table 1**.

**Table 1:** Amino acid sequence of mutated COMP P9 peptides.

Peptide	Amino acid sequence
P9-human	**APGFCFPGVACIQTESGA**
P9-1	**APGSCFPGVACIQTESGA**
P9-2	**APGFCFPGVVCIQTESGA**
P9-3	**APGFCFPGVACSETESGA**
P9-4	**APGFCFPGVACIQTASGA**

Red font means the mutated amino acid site compared with P9-human.

#### Synthetic Cyclic Peptides

Synthetic cyclic peptides were synthesized as previously described (24).

#### Mutated COMP_F95S

For the expression of a single amino acid mutated COMP_F95S, two primers were designed with forward primer: 5’- AGGTTCCTGCTTTCCCGGAGTAGCG-3’ and reverse primer: 5’-GGTGCACAGTGCAGCAGAGGCC-3’. A Q5 site-directed mutagenesis kit (E0554S, New England BioLabs) was used according to the manufacturer’s instruction. After transformation, several colonies were selected and the sequences verified. The plasmid with the right sequence was transfected into the Expi393FTM cells as described above.

### Induction of Experimental Arthritis

Mice were immunized intradermally (i.d.) at the base of the tail with 100 ug of native COMP emulsified 1:1 in Freund,s complete adjuvant (CFA, #263810, Difco, BD) or in Freund,s incomplete adjuvant (IFA, #263910, Difco, BD) containing Mycobacterium Tuberculosis H37Ra (M.T., #231141, Difco, 0.5 mg/ml) in a total volume of 100 ul on day 0. All the mice were boosted on day 35 with 50 ug of native COMP in IFA in a total volume of 50 ul. Mice were double blind scored 3 times per week for the peripheral joint inflammation, starting 2 weeks after the first immunization. The evaluation of arthritis was conducted in a blinded manner according to an extended scoring protocol (25), using 0–15 scale for each paw (0 = normal joint; 1 = each swollen toe or joint; 5= one swollen ankle). The total maximum score per mouse was 60 points.

To evaluate the requirement of the native structure of COMP for the arthritis induction, mice were immunized with 100 ug of native COMP (group 1), denatured COMP (group 2) emulsified 1:1 in Freund^’^s complete adjuvant (CFA, #263810, Difco, BD) or CFA only (group 3) separately on day 0. On day 35, all the mice were boosted with or without COMP in IFA (#263910, Difco, BD) in a total volume of 50ul (group 1 = 50 ug of native COMP in IFA; group 2=50ug of denatured COMP in IFA; group 3 = IFA only). The evaluation of arthritis was conducted as described above.

To prove the importance of phenylalanine at position 95 for the T cell epitope recognition and arthritis development, mice were immunized i.d. at the base of the tail with 100ug of COMP_F95S or native COMP emulsified 1:1 in Freund’s complete adjuvant (CFA, #263810, Difco, BD) on day 0. On day 35, the mice were boosted with 50ug of COMP_F95S or native COMP in IFA (#263910, Difco, BD) in a total volume of 50ul. The evaluation of arthritis was conducted as described above.

### Detection of Autoantibodies

Detection of serum levels of antibodies against COMP and CII: Serum antibody levels against COMP were analyzed in 96-well plate (Nunc, Thermo Fisher Scientific, Denmark) by ELISA using recombinant human COMP. Recombinant human COMP (50uL/well; 10ug/ml in phosphate buffer saline (PBS), PH 7.4) was precoated overnight at 4. Then the plates were blocked with 3% milk in PBS to decrease background disturbance. The plates were washed for 5 times with ELISA buffer (PBS with 0.1% Tween 20). The serum was diluted (*500) in PBS (5*time titration, 5 wells for each sample), and incubated for 2 h. Serum from COMP- induced arthritis mice were used as positive control. 50ul of secondary antibody was added and incubated for 1 h. The secondary antibodies were as following: Rat Anti-Mouse Kappa- Horseradish peroxidase (HRP) (Southern Biotech, #117-05), Goat Anti-Mouse IgG(H+L) (Southern Biotech, #1031-05), Goat Anti-Mouse IgG_1_ (Southern Biotech, #1070-05) and Goat Anti-Mouse IgG_2b_-HRP (Southern Biotech, #1091-05). After washing for 5 times, ExtrAvidin^®^ -Peroxidase (1:5000 dilution, Sigma E-2886) was added and incubated for 45 mins. 5mg ABTS substrate (ABTS tablets, Boehringer Mannheim) was diluted in 5 ml of freshly prepared buffer (ABTS buffer, Boehringer Mannheim). After 20 min-incubation in the dark at 20, absorbance was measured at 405nm (OD405). Serum antibody against CII were analyzed by sandwich ELISA, as previously described (26).

### Immune Assays

#### Peptide-Based ELISA

In the first experiment, we performed epitope mapping of whole human COMP by ELISA to identify linear B cell epitopes with serum from COMPIA B6N model. Maxisorb plates were coated with COMP peptides or native COMP at a concentration of 5 ug/ml in PBS overnight at 4. The second day the plates were washed with PBST for 3 times and then blocked with 1% bovine serum albumin (BSA) in PBS. Different dilutions (1:100 to 1:2500) of mice serum (B6N mice immunized with COMP+CFA) were added to the wells and incubated for 1 h at room temperature. Then the plates were washed with PBST for 3 times. HRP-conjugated anti-mouse second antibody was added for further incubation of 1 h followed by the detection using ABTS-HRP system as previous description. Absorbance was measured at 405nm (OD405).

#### ELISpot Assay

The 96-well ELISpot plates (MSIPS4W10, Millipore) were prewetted with 70% EtOH and then precoated with anti-mouse IFN-γ capture antibody R46A2 (15 ug/ml, 50 ul/well) overnight at 4. The second day the plates were washed with PBS for twice and then blocked with 1% BSA in PBS. Splenocytes or cells from draining lymph nodes were obtained from the immunized B6N mice and added to the plates (1x10^6^ cells per well for splenocytes, 0.5x10^6^ cells per well for lymph node cells). The cells were stimulated with 50 ug/ml different peptides (peptides from human COMP library; P9; P9-1, P9-2, P9-3, P9-4) in triplicates at 37for 24 h. Concanavalin A (Con A, 1 ug/ml) used as a positive control. The plates were washed with PBS twice and a washing buffer (PBS containing 0.01% Tween 20) for 4 times followed by incubation with 50ul/well of biotinylated anti-IFN-γ (clone An18, 4 ug/ml in PBS/BSA 0.5%) at room temperature for 2 h. After washing the plates with the washing buffer for 5 times, streptavidin-alkaline phosphatase (50 ul/well, 1:2500 in PBS) was added to each well and incubated for 45 min at room temperature. After washing with the washing buffer for 3 times and PBS for 3 times, cytokine spots were visualized using Sigma Fast BCIP/nitroblue tetrazolium (100 ul/well) and enumerated using an ImmunoScan ELISpot Analyzer (CTL Europe).

### Histological Analyses

Mice were sacrificed at the end time point, and the hind paws were dissected and subsequently fixed in 10% buffer formalin solution. Fixed tissues were decalcified for 30 days in EDTA-solution (100g EDTA, 75 g polyvinylpyrrolidone, Tris 0.1M in 1L H_2_O, add KOH to PH = 6.95) for decalcification, followed by dehydration and paraffin embedding. Tissue sections (7 μm) were then stained with hematoxylin and eosin (HE). HE-stained sections were blindly assessed in naive B6N mice or B6N mice immunized with native COMP.

### Molecular Modeling

The homology model of the MHC class II molecule A^b^ in complex with COMP (92–109) was constructed using the SWISS-MODEL (27), and the crystal structure of A^b^ with an PADI4 peptide (PDB code: 6MNM) was selected as a template due to high sequence similarity (28). The potential core binding residues (FCFPGVACI) of COMP (92–109) was predicted using Consensus algorithm (29). For modeling the COMP (92–109) peptide in the binding groove of A^b^, the PADI4 peptide from crystal structure of A^b^ in complex with PADI4 peptide (PDB code: 6MNM) was used as a basis, then several residues were modeled by FoldX program (30), then the side chains were optimized by Swrl4 program (31).

### Statistical Analyses

All data are expressed as the mean ± SEM. Mann-Whitney test and Kruskal-Wallis test were used for statistical analyses (GraphPad Prism version 8.0.2). *p*<0.05 was considered as significant *p<0.05, ** *p*<0.01.

## Results

### Immunization With COMP Induces Severe Arthritis With High Incidence in B6N Mice

**Figure 1:**
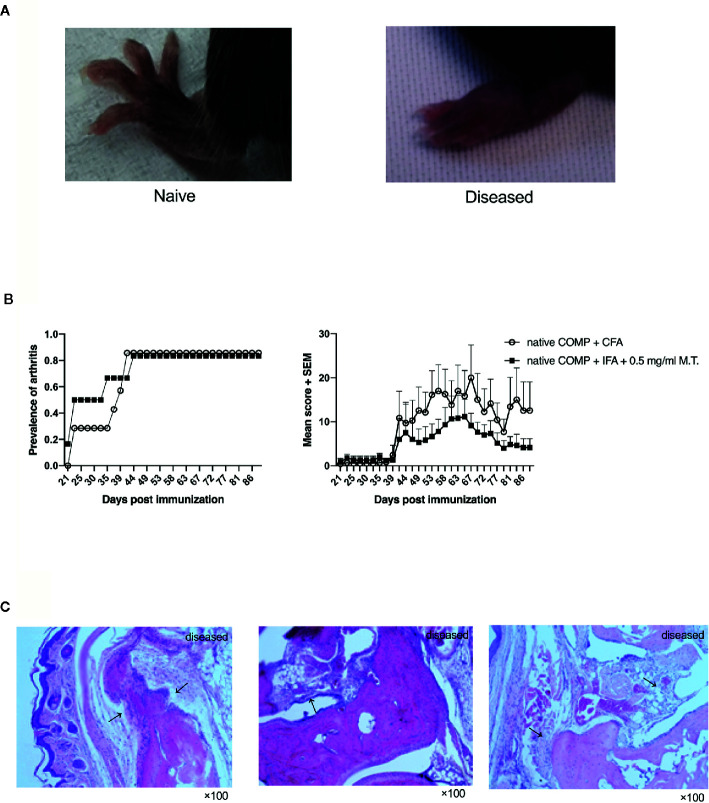
Native COMP induces arthritis in B6N mice with high incidence and severe arthritis. **(A)** The arthritis joint phenotype of B6N mice. **(B)** B6N mice were immunized with native COMP emulsified in CFA (n=7) or IFA+0.5 mg/ml M.T. (n=6), and monitored for prevalence and clinical score of arthritis. **(C)** Histological analysis of ankle joint of B6N mice with COMP-induced arthritis at the end of the experiment. Sections were stained with H&E staining. Prominent synovial hyperplasia, inflammatory cells infiltration and cartilage and bone destruction in mice immunized with native COMP+CFA. All images were taken at ×100 magnification.

To test the potential of COMP for the induction of arthritis, we immunized B6N mice with COMP emulsified in complete Freund, adjuvant (CFA, #263810, Difco, BD) or in incomplete Freund’s adjuvant (IFA, #263910, Difco, BD) containing Mycobacterium Tuberculosis strain H37Ra (M.T., #231141, Difco, 0.5 mg/ml). Both groups immunized with COMP developed severe arthritis with high incidence (**Figures 1A, B**).

The arthritic joints from arthritic B6N mice revealed prominent synovial hyperplasia, inflammatory cells infiltration and cartilage and bone destruction in both group of mice immunized with native COMP+CFA or native COMP + IFA + 0.5 mg/ml M.T. at the end time point of the experiment, similarly to human RA (**Figure 1C**).

### Native COMP Induces Antibody Responses and Specific B Cell Epitopes Are Recognized in B6N Mice

**Figure 2:**
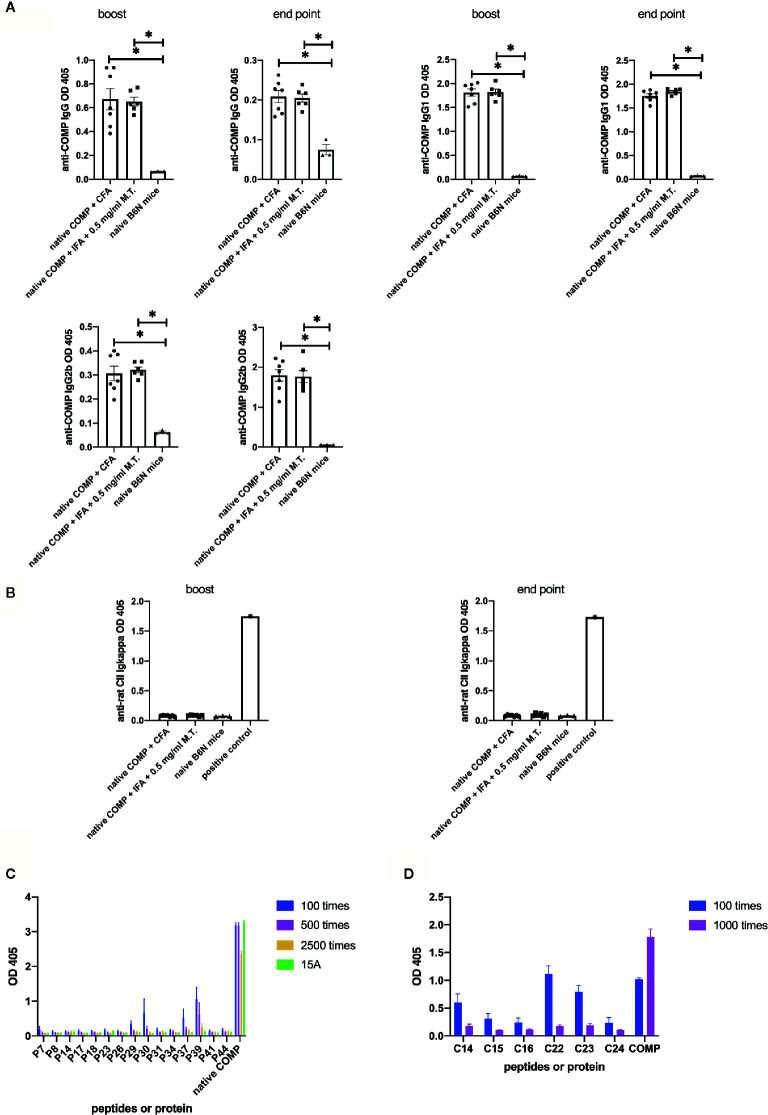
Native COMP induces antibody responses and specific B cell epitopes are characterized in B6N mice. **(A, B)** Native COMP induced anti-COMP antibodies but no anti-CII antibodies in B6N mice. **(A)** Both native COMP+CFA group (n=7) and native COMP+IFA+0.5 mg/ml M.T. group (n=6) have anti-COMP IgG, gG1 and IgG2b production throughout the experiment (*p < 0.05). There are no differences of anti-COMP IgG, IgG1, and IgG2b levels between the two groups (*p*>0.05). **(B)** No response to rat CII could be detected in COMP immunized mice (*p*>0.05) (A B6N mouse immunized with rat CII was chosen as positive control). **(C)** Mapping of human COMP linear B-cell epitopes using overlapping peptide library of COMP protein (n=6) **(D)** According to the results of experiment A **(C)**, We choose six cyclic COMP peptides (C14, C15, C16, C22, C23 and C24) in a cyclic COMP library, which are similar to the sequences of the candidate peptides (peptide 7, 29, 30, 37, 39). We precoated the plate with these cyclic peptides and added two dilutions of serum of B6N mice immunized with COMP with 2 dilute (diluted 100 times and 1000 times) to test the OD value by ELISA. Positive binding was defined as 3SD from the mean of naïve mice (n=4). Antibody binding was measured by ELISA. The results presented as absorbance at nm 405.

Next, we compared anti-COMP autoantibody responses between three groups. Mice in both immunized groups (immunized with native COMP+CFA or native COMP + 0.5 mg/ml M.T.+IFA) developed a prominent IgG anti-COMP autoantibody response, compared with naïve B6N mice (*p<0.05). There was no difference between mice immunized with native COMP+CFA and mice immunized with native COMP + 0.5 mg/ml M.T.+IFA (*p*>0.05) (**Figure 2A**). Similar trends were observed in values of IgG1 and IgG2b between three groups (**Figure 2A**).

To test whether an immune response to CII was triggered, we analyzed anti-CII Ig levels. Anti-CII Ig levels, as detected with antibodies to kappa, showed no significant difference between immunized and naïve B6N mice (**Figure 2B**).

To confirm that the B cell response is specific for COMP and to identify peptide B cell epitopes, we first screened an overlapping peptide library of COMP with serum from COMP-immunized B6N mice by ELISA. Serum samples were taken at the end point. Results indicated five potential B cell epitopes (peptide 7, 29, 30, 37, 39). We selected 15 peptides (including the five potential epitopes) to repeat the experiment at different serum dilution (n=4), which confirmed the result (**Figure 2C**). We subsequently selected six cyclic COMP peptides with the same sequences as the candidate peptides and used them for testing the serum response. It confirmed that these peptides had positive reaction with immunized mice serum (**Figure 2D**), showing that these are peptide sequential epitopes. In conclusion, a peptide specific antibody response to COMP is induced after immunization of COMP in the B6 mouse.

### Induction of Arthritis Is Dependent on the Native Structure of COMP

**Figure 3:**
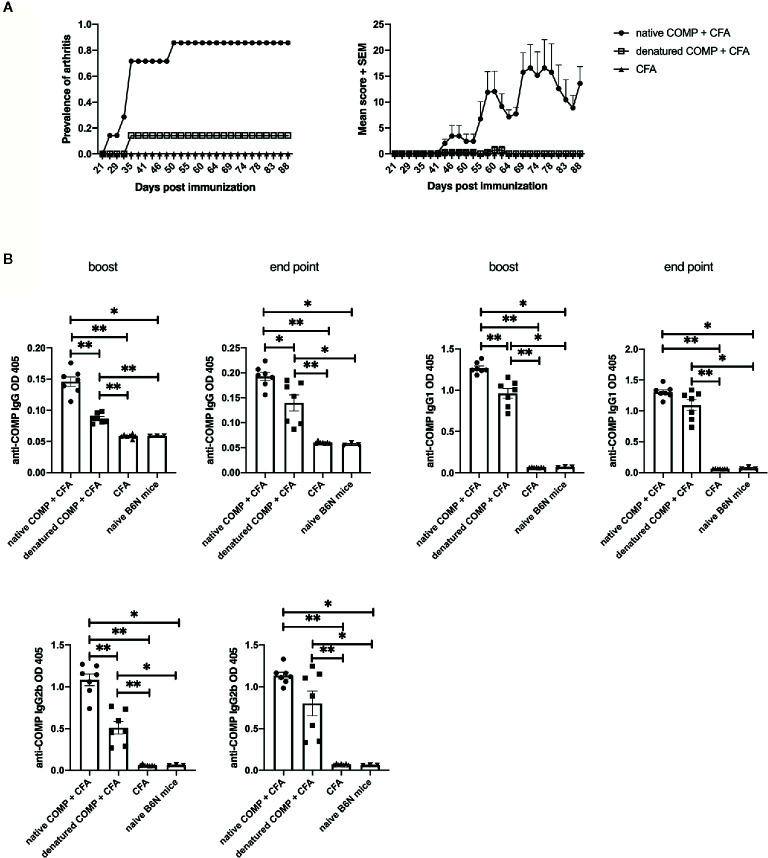
Induction of arthritis requires COMP to be in its native conformation. **(A)** B6N mice were immunized with native COMP + CFA (n=7), denatured COMP + CFA (n=7) or CFA only (n=7) and monitored for prevalence and clinical score of arthritis. Left panel shows immunization with native COMP can induce arthritis in B6N mice with high incident rate (100%); however, immunization with denatured COMP can induce arthritis in B6N mice with very low incident rate (14.3%); immunization with CFA only do not induce arthritis. Right panel shows the clinic score of the three groups. **(B)** Immunization with native COMP induces a stronger specific IgG response than immunization with denatured COMP (*p < 0.05, **p < 0.01). Blood were collected 35 days (boost) after the immunization and the end point from B6N mice. Filled circles indicate mice immunized with native COMP + CFA; filled squares indicate mice immunized with denatured COMP + CFA; filled triangles indicate mice immunized with CFA only; inverted filled triangles indicate naïve mice.

To investigate if the conformational structure of COMP has an effect on arthritogenicity, we immunized B6N mice with native COMP + CFA, denatured COMP + CFA or CFA separately. All B6N mice immunized with native COMP developed severe arthritis whereas mice immunized with denatured COMP developed only very mild arthritis with low incidence (14%). As expected, B6N mice immunized with CFA did not develop arthritis (**Figure 3A**).

To test whether the native conformational of COMP is recognized by antibodies developed after the immunization with both COMP preparations, we analyzed the sera at different time points during COMPIA (at days 35 and 88 post-immunization). Both native COMP immunized group and denatured COMP group developed an anti-COMP antibody response at both time points (*p<0.05) (**Figure 3B**). However, the immunization with native COMP induced stronger IgG, IgG1, and IgG2b anti-COMP responses than immunization with denatured COMP (*p<0.05). We conclude that antibodies to conformational epitopes are pathogenic and need for the induction of arthritis and that these antibodies are likely triggered by help from COMP specific T cell.

### Identification of a Major T Cell Epitope, of Critical Importance for Development of Arthritis

**Figure 4:**
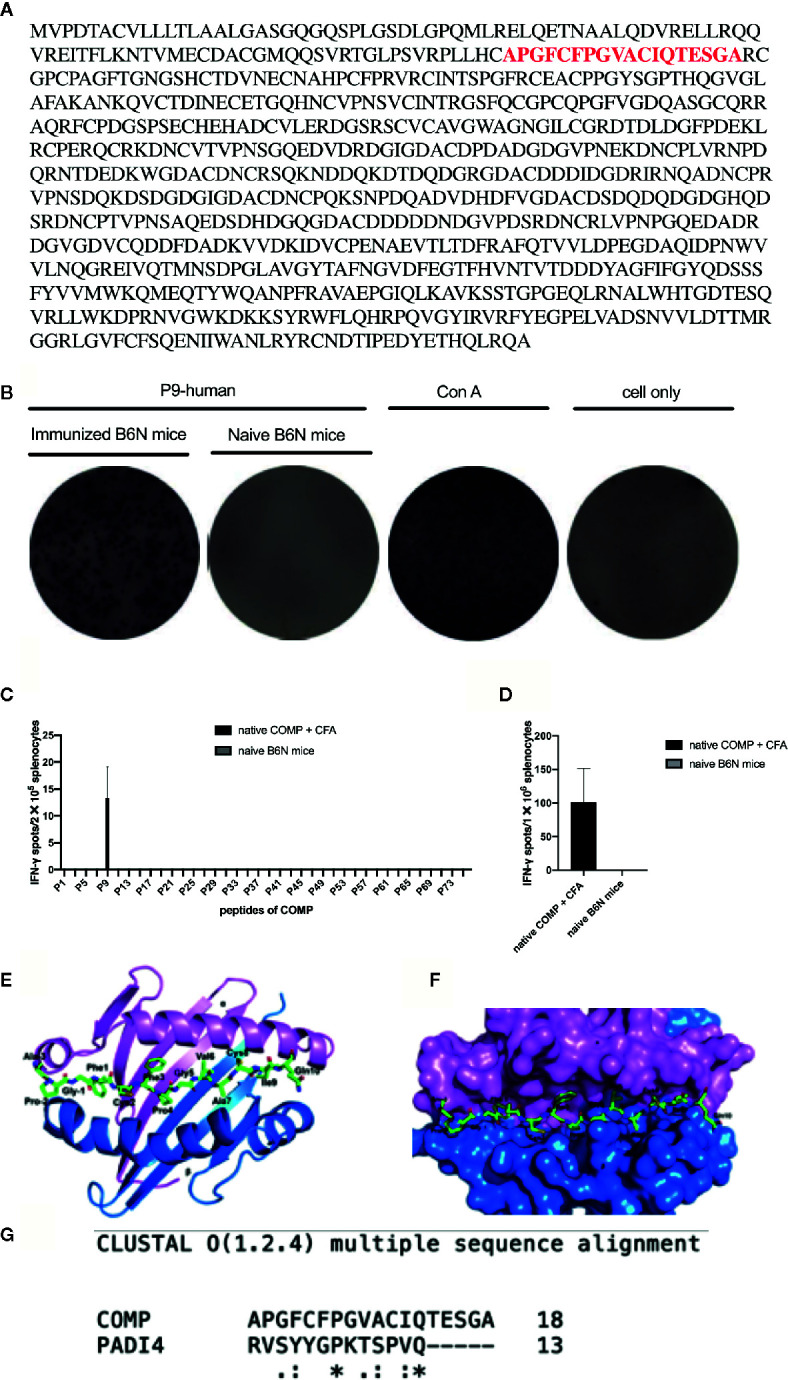
P9 (human COMP peptide 92-109) is identified as the key T cell epitope, which induces arthritis in COMP-immunized B6N mice. We build an overlapping peptides library of human COMP, which includes 76 peptides. **(A)** shows the human COMP sequence and the red sequence is the candidate T cell epitope. **(B)** shows that P9 of human COMP shows positive T-cell reaction in COMP-immunized B6N mice. **(C)** peptides screening: B6N mice were immunized with native COMP + CFA (n=3), and naïve B6N mice (n=3) were chosen as control. 14 days later, splenocytes were harvested, 2x10^5^ cells were added to each well (50 ug/ml for peptide, 76 peptides for each well separately) for 24 h in an IFN-γ ELISpot assay to screen the COMP library. P9 shows positive T-cell reaction in COMP-immunized B6N mice, which suggests it should be the candidate T cell epitope. **(D)** P9 identification: B6N mice were immunized with native COMP + CFA (n=3), and naïve B6N mice (n=3) were chosen as control. 14 days later, splenocytes were harvested, 1x10^6^ cells were incubated with P9 (50 ug/ml) for 24 h in an IFN-γ ELISpot assay. The peptide with spots >10 with PP/control ratio ≥ 3 is considered as the positive peptides. Graph shows the mean number of spots ± SEM from 3 individual B6N mice. **(E)** Top view of COMP- P9 - H-2^b^ shown in cartoon presentation with the peptide (P9) shown in stick representation. α, β of H-2^b^ and COMP-P9 are colored with pink, blue and green, respectively. **(F)** Proposed model of overall crystal structure of H-2^b^/P9 peptide complex: P9 peptide recognition by the H-2^b^ loop. α, β of H-2^b^ and COMP-P9 are colored with pink, blue and green, respectively. **(G)** The P9 peptide structure modeled based on PADI4 structure.

A library of overlapping human COMP peptides (Fig 4A) were tested for their ability to activate T cells after immunization with human COMP. IFN-γ secretion by CD4^+^ T cells was used as a read-out and ConA was used as a positive control. Spleen cells from mice immunized with COMP showed strong IFN-γ T cell recognition of peptide P9 (APGFCFPGVACIQTESGA) (**Figure 4A**). Out of 76 pairs of peptides tested, only P9 was able to significantly active CD4+T cells from H-2^b^ mice (**Figures 4B–D**). Additionally, splenocytes from naïve B6N mice were tested, which shows negative reaction to P9 (**Figures 4B–D**).

To determine how the P9 peptide is located in the Ab MHC class II molecule we compared with earlier published structural data (32). The topological structure of the binding of the P9 peptide fitted will molecular modeling and docking onto the Ab molecule (**Figure 4E**). On the A^b^ structure, COMP-P9 fits into the most regions of the A^b^ groove (**Figures 4F, G**). We conclude that the A^b^ can accommodate the P9 peptide which adopts an MHC class II-binding conformation.

### Identification of Critical Amino Acids Within the Immunodominant COMP Epitope

**Figure 5:**
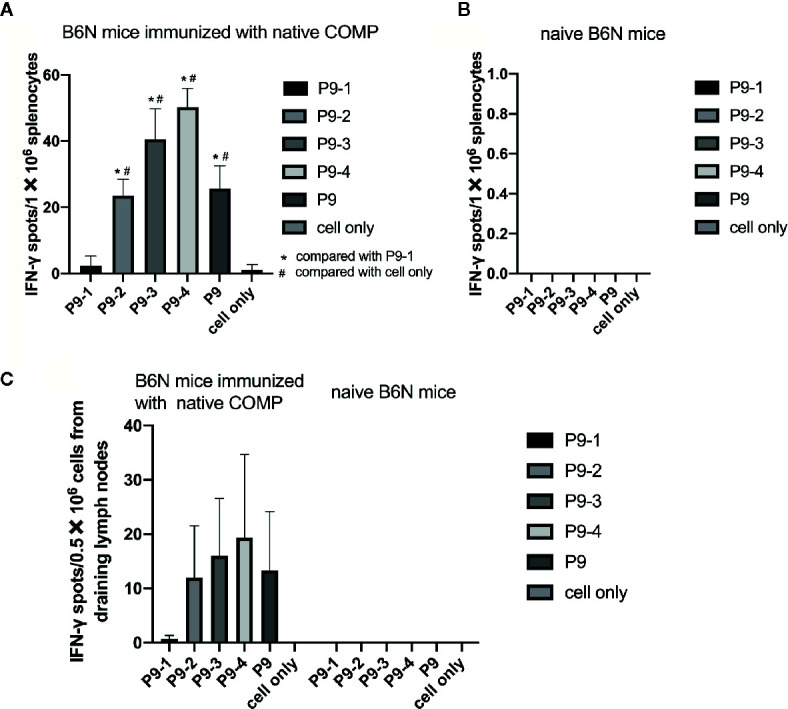
Identification of the critical amino acids of the P9 peptide. **(A, B)** T cell response against different mutated peptides or control as detected in IFN-γ ELISpot assay after 14-day recall stimulation. Splenocytes of COMP immunized B6N mice (n=3) were stimulated with P9 or different mutated peptides (P9-1, P9-2, P9-3, P9-4), followed by ELISpot assay to determine the number of IFN-γ spot. Naïve B6N mice (n=3) were used as control group. Con A were included as positive control. **(C)** Lymph node cells of COMP immunized H-2^b^ mice were stimulated with P9 or different mutated peptides (P9-1, P9-2, P9-3, P9-4), followed by ELISpot assay to determine the number of IFN-γ spot. Histogram plots present data of 3 B6N mice immunized with native COMP. The control conditions are lymph node cells from naïve B6N mice stimulated with the peptides above. Positive T cell responses were defined as SPU/0.5×10^6^ cells ≥ 10 with PP/control ratio ≥ 3. *p < 0.05 compared with P9-1, ^#^ *p* < 0.05 compared with cell only (no peptide).

To make a strong T cell recall response to human COMP the targeted peptide must differ from the endogenous peptide, to avoid tolerance. To identify the critical amino acids, we compared the sequence of mouse COMP with human COMP and found differences at several positions. New peptides varying at these positions were synthesized (**Table 1**) and tested in the IFN-γ ELISpot assay using splenocytes of human COMP immunized B6N mice (**Supplement Figure 2**). We observed that P9-2, P9-3, P9-4 and P9 induced IFN-γ secretion, but not P9-1 (**Figures 5A–C**). This suggest that the phenylalanine at position 95 is critical for the immunogenicity of human COMP. Imaging analysis of the peptide interaction with MHC class II showed that this amino acid is most likely filling the P1 position on the A^b^ molecule (**Figure 4E**).

### Phenylalanine at Position 95 of COMP Is Critical for Induction of Arthritis

**Figure 6:**
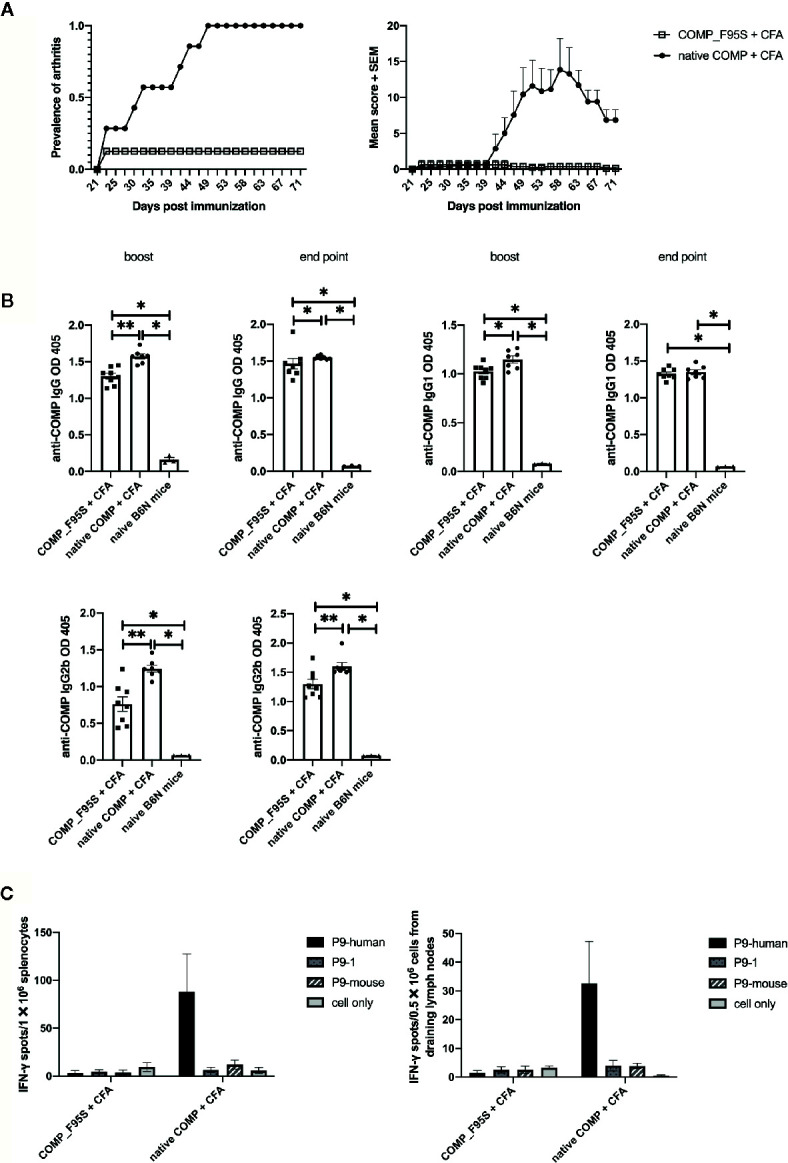
Only COMP, and not COMP_F95S, induces severe arthritis. **(A)** B6N mice were immunized with native COMP (n=7) or COMP_F95S (n=8) emulsified in CFA and monitored for development of arthritis. Left panel shows the prevalence of arthritis; Right panel shows clinical scores of arthritis. The figures show that immunization with native COMP induce severe arthritis in 7 out of 7 B6N mice whereas only 1 out of 8 mice immunized with COMP_F95S developed arthritis. **(B)** B6N mice were immunized with native COMP + CFA (n=7) or COMP_F95S + CFA (n=8). Blood were collected 35 days after the immunization and at the end point. Filled circles indicate mice immunized with native COMP + CFA; filled squares indicate mice immunized with COMP_F95S + CFA; filled triangles indicate naïve mice. The antibody levels (IgG, IgG1 and IgG2b) are higher in native COMP + CFA group than COMP_F95S + CFA mice (*P < 0.05, **P < 0.01). **(C)** Mice immunized with COMP_F95S have no reaction with P9 of COMP in T cell recall assay. Left panel: 14 days later, splenocytes of COMP_F95S (n=3) or native COMP (n=3) immunized mice were stimulated with peptide (P9-human, P9-1 and P9-mouse) or protein (COMP_F95S and native COMP), followed by ELISpot assay to determine the number of IFN-γ spot. It shows that splenocytes of mice immunized with native COMP has strong T cell reaction with P9 of human COMP, however, splenocytes of mice immunized with COMP_F95S has no reaction with P9 of human COMP. Right panel: 14 days later, cells from draining lymph nodes of COMP_F95S (n=3) or native COMP (n=3) immunized mice were stimulated with peptide (P9-human, P9-1 and P9-mouse) or protein (COMP_F95S and native COMP), followed by ELISpot assay to determine the number of IFN-γ spot. It shows that cells from draining lymph nodes of mice immunized with native COMP has strong T cell reaction with P9 of human COMP, however, cells from draining lymph nodes of mice immunized with COMP_F95S show no reaction with P9 of human COMP. Positive T cell responses were defined as SPU/0.5 × 10^6^ cells ≥ 10 with PP/control ratio ≥ 3.

To determine whether phenylalanine at position 95 is critical for the development of arthritis we made a new recombinant COMP protein replacing phenylalanine with serine at position 95 (COMP_F95S). B6N mice were immunized with COMP or COMP_F95S in CFA. As expected all mice immunized with COMP developed severe arthritis. Only 1 out of 8 B6N mice immunized with COMP_F95S developed arthritis, with very low scores (**Figure 6A**).

To test whether COMP_F95S immunization induces anti-COMP antibodies, we determined the production of anti-COMP antibodies during the disease progression. The antibody levels (IgG, IgG1, and IgG2b) were clearly higher in mice immunized with native COMP group compared with those immunized with COMP_F95S group at both day 35 and at the endpoint (**Figure 6B**).

We tested the T cell response specificity in a recall IFN-γ ELISpot assay (**Supplement Figure 3**). As expected the COMP immunized mice showed a response to the P9 peptide but not the COMP_F95S immunized mice. Interestingly, mice immunized with COMP_F95S could still make a detectable T cell response to COMP protein indicating that the F95S mutation did not abolish all T cell responses, only the T cell response of importance for arthritis development (**Figure 6C**).

## Discussion

In this study, we show that human COMP, a non-collagen glycoprotein primarily produced by cartilage, can induce arthritis in C57BL/6 mice that carry the H-2^b^ haplotype, which is useful as a model for human RA. It requires the T cell recognition of a COMP derived peptide bound to the A^b^ molecule.

The new COMPIA model in B6N mice has many similarities with the well-known CIA model. It induces a severe polyarthritis in diarthrodial joints. It is MHC class II associated and the COMP-derived peptide recognized by T cells is defined. In similarity with CIA the induction of arthritis is facilitated by a T cell response to non-self -COMP, due to that the immunodominant peptide is not binding well to the MHC class II molecule and is thus likely to be ignored for development of tolerance (33). The COMP immunization also induces a strong antibody response, which likely contributes to the development of arthritis (34). Importantly, the COMPIA model has a major advantage as compared with the CIA model, it is inducible in C57BL/6 mice, carrying the H-2^b^ haplotype. This is important as C57BL/6 mice is regarded as a standard background in immunology research and is a background commonly used for genetically modified mouse strains. Thus, to use the novel COMPIA model in B6 mice does not require the time and resources needed for backcrossing of mice to introgress a proper MHC class II haplotype, as the MHC class II molecule A^q^ is needed to make a proper CIA experiment (8, 13). Immunization with CII in the B6 mice may lead to arthritis, but the triggering of an immune response is due to T cell recognition of a contaminant, most likely complexed with CII. COMPIA can be induced with recombinant COMP and is dependent on a specific T cell response to COMP. Interestingly, in similarity with CII, COMP induces arthritis in mice expressing the murine A^q^ and the human DR*0401 MHC class II molecules (18). Thus, the COMPIA model is useful in many strains developed for arthritis research and the immune response is directly comparable with human RA.

Systemic delivery of nanoparticles coated with antigens (autoimmune-relative peptides) bound to MHC class II molecules trigger autoimmune reaction in different mouse models (35), which confirms the core role of peptide recognition by MHC class II in the pathogenesis of autoimmune diseases. Recognition of peptide by an H-2^b^ derived MHC class II molecule has not earlier been reported to be of relevance in a RA model. To understand the detailed molecular basis of the interaction of COMP-P9 with H-2^b^, we mutated the amino acids of COMP-P9 and produced the mutated protein by refolding Escherichia coli, we demonstrated a direct interaction between COMP-P9 with the A^b^ molecule. The mutational experiment resulted in a different profile of arthritis susceptibility, as the mutants COMP (COMP-F95S) resulted in eight-time reduction of incidence of arthritis. Our experiments provide a conclusive biochemical evidence that the A^b^ molecule binds COMP-P9 leading to COMP-specific T cell response in B6N mice, that is a key step for induction of arthritis.

In summary, a new model for RA has been established using C57BL/6 (H-2^b^) mice through immunization with COMP, which is dependent on a COMP specific peptide, thus in similarity with collagen induced arthritis in H-2^q^ (A^q^) expressing strains.

## Data Availability Statement

The raw data supporting the conclusions of this article will be made available by the authors, without undue reservation.

## Ethics Statement

The animal study was reviewed and approved by Stockholm regional ethics committee for animal research, Sweden (N35/16).

## Author Contributions

YZ was in charge of the animal experiments, methodology, software, formal analysis, resources, investigation, data collection, and wrote and edited the original draft. VU conducted the investigation and methodology. BX conducted the investigation and methodology. WC conducted the investigation. CG conducted the investigation, and provided the methodology and software. ZS conducted the investigation. RH conceptualized the study and subject design, wrote, reviewed, and edited the manuscript, provided the resources, and supervised the study. All authors contributed to the article and approved the submitted version.

## Funding

This work was supported by grants from the Swedish Research Council (2015-02662), Knut and Alice Wallenberg Foundation (KAM2015.0063), the Swedish Foundation for Strategic Research (RB13-0156) and Science and Technology Projects of Guangzhou (Grant No. 201707010365).

## Conflict of Interest

The authors declare that the research was conducted in the absence of any commercial or financial relationships that could be construed as a potential conflict of interest.

## Abbreviations

CIA, collagen-induced arthritis; COMP, cartilage oligomeric matrix protein; RA, rheumatoid arthritis; CII, type II collagen; SPF, specific pathogen-free; CFA, complete Freund adjuvant; IFA, in incomplete Freund^’^s adjuvant.
