# Supplementary figures and images for: Cartilage Oligomeric Matrix Protein Induced Arthritis—A New Model for Rheumatoid Arthritis in the C57BL/6 Mouse
Source: Front Immunol. 2021 Feb 23;12:631249. doi: 10.3389/fimmu.2021.631249 (PMC7940517; doi:10.3389/fimmu.2021.631249)

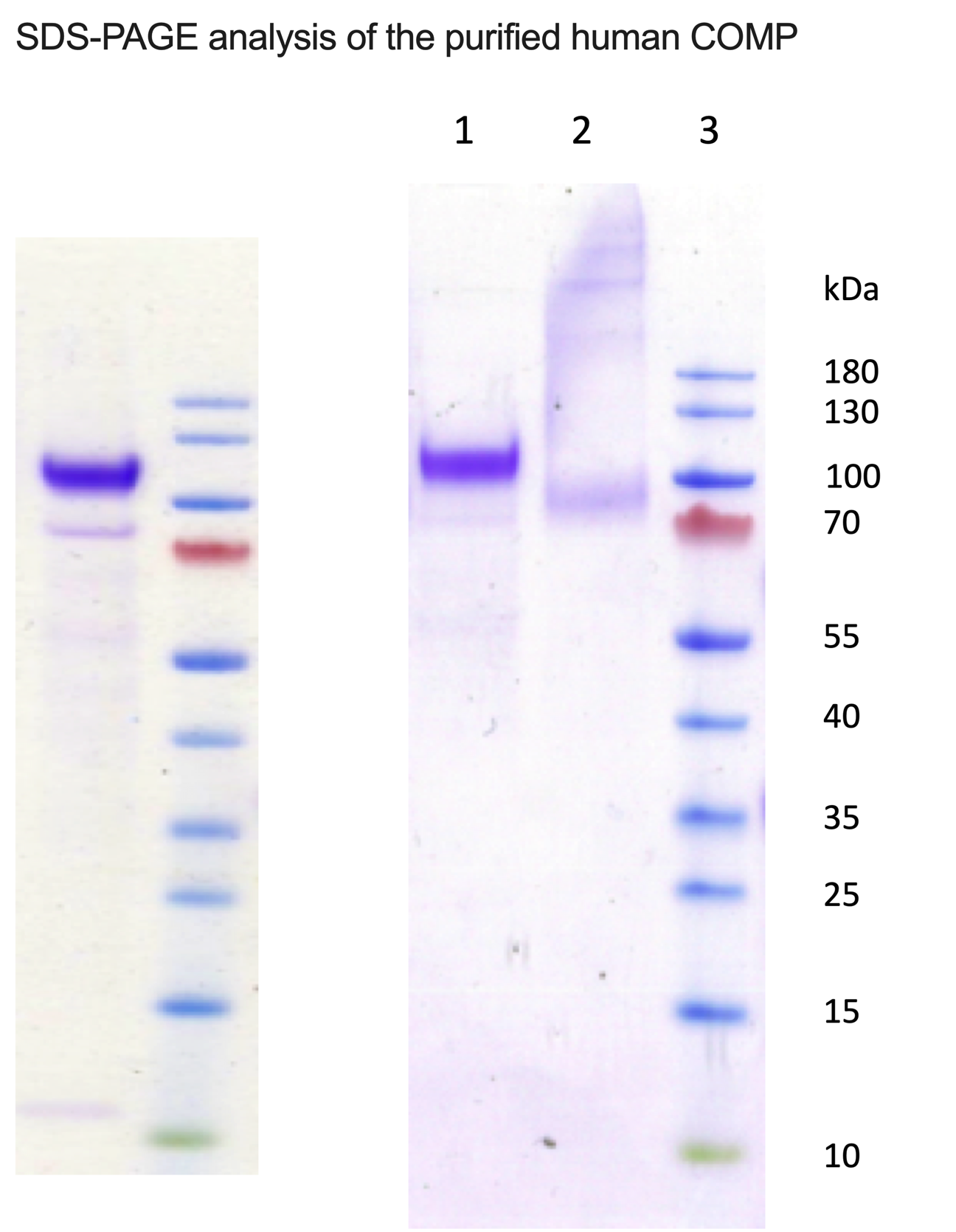

Supplement: Supplementary file 1 [file Image_1.tiff]

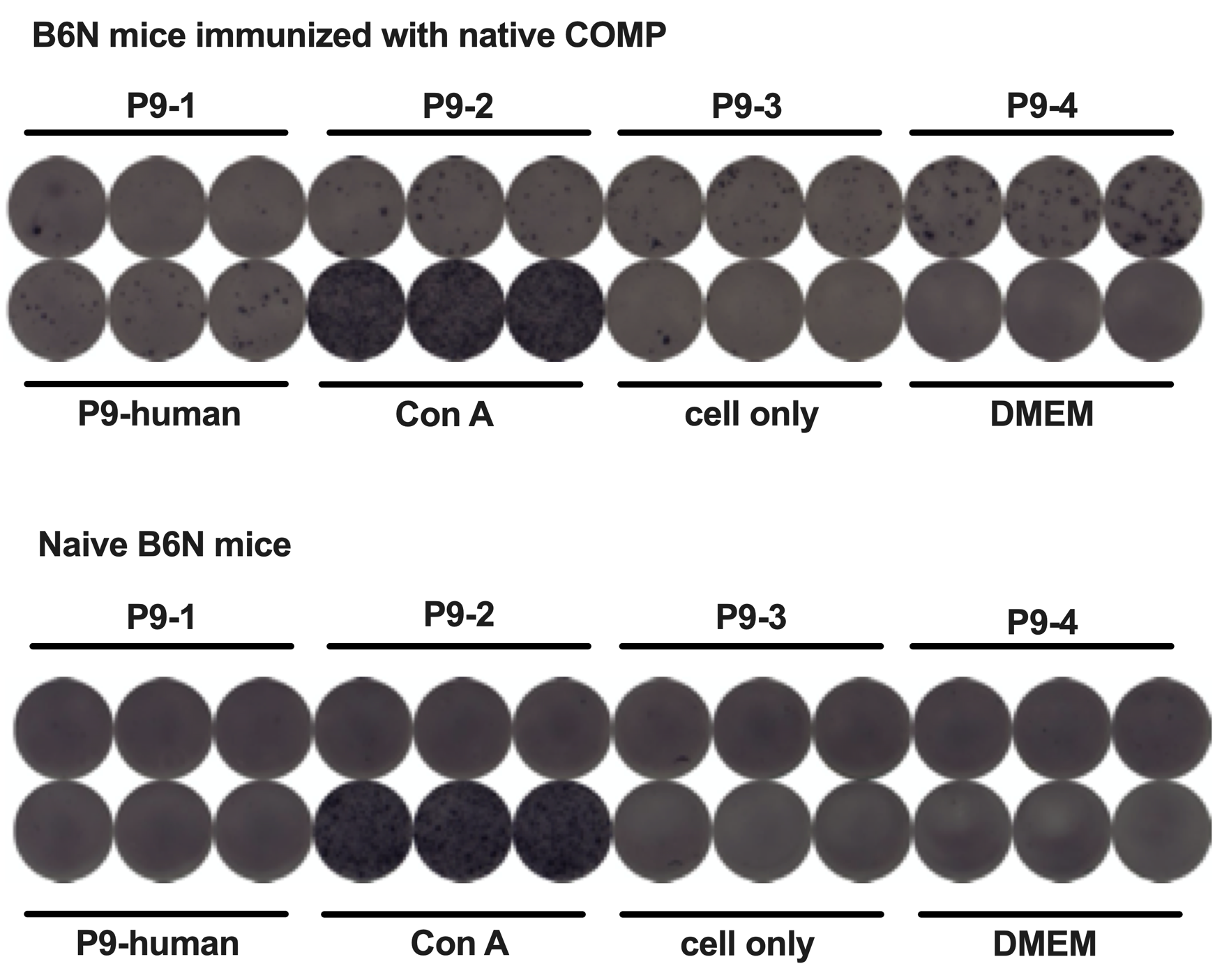

Supplement: Supplementary file 2 [file Image_2.tiff]

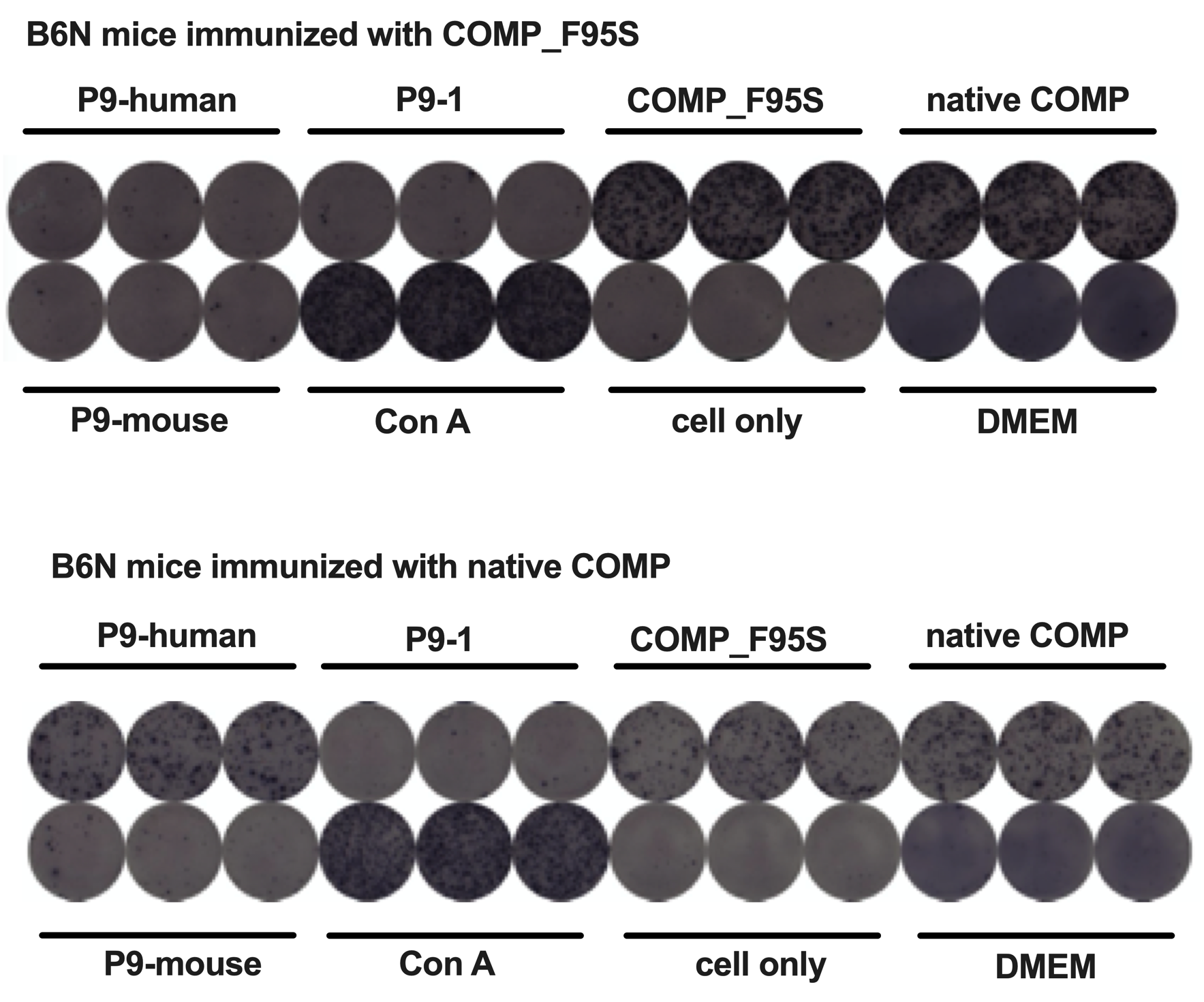

Supplement: Supplementary file 3 [file Image_3.tiff]
